# Uncovering the secret life of Rho GTPases

**DOI:** 10.7554/eLife.53276

**Published:** 2019-12-13

**Authors:** Jenna A Perry, Amy Shaub Maddox

**Affiliations:** Department of BiologyUniversity of North Carolina at Chapel HillChapel HillUnited States

**Keywords:** RhoGTPase, RhoGDI, Cdc42, Rho, *Xenopus*

## Abstract

New methods to directly visualize Rho GTPases reveal how a protein called RhoGDI regulates the activity of these 'molecular switches' at the plasma membrane.

**Related research article** Golding AE, Visco I, Bieling P, Bement WM. 2019. Extraction of active RhoGTPases by RhoGDI regulates spatiotemporal patterning of RhoGTPases. *eLife* **8**:e50471. doi: 10.7554/eLife.50471

Cells are busy places that are constantly responding to a variety of internal and external demands. Molecules that can switch back and forth between two or more states have an important role in helping cells respond to these demands. An important type of molecular switch is the Rho family of GTPases: these proteins cycle between an active form (in which the GTPase is bound to guanosine triphosphate) at the cell membrane and an inactive form (in which it is bound to guanosine diphosphate) that is thought to only be found in the cytoplasm (Figure 1).

**Figure 1.**
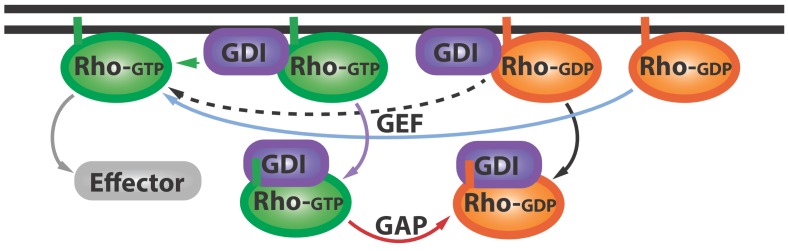
The Rho GTPase cycle. Rho GTPases cycle between active (Rho-GTP; green) and inactive (Rho-GDP; orange) forms: the inactive form is activated (blue arrow) by proteins called GEFs (not shown), and the active form can go on to activate (grey arrow) various downstream effector proteins (grey). Other proteins called GDIs (purple) can bind inactive Rho GTPases and extract them from the membrane (black arrow). Now Golding et al. have shown that GDIs can also bind to active Rho GTPases and extract them from the membrane (purple arrow). Proteins called GAPs (not shown) help convert these active GDI-Rho-GTP complexes into inactive GDI-Rho-GDP complexes (red arrow): this can happen at the membrane or in the cytoplasm. Further research is needed to address a number of questions: can Rho-GTP escape from the GDI-Rho-GTP complex (green arrow)? And how can GEFs activate the Rho-GDP in GDI-Rho-GDP complexes (dashed black arrow)? GEF: guanine nucleotide exchange factor; GDI: Guanine nucleotide dissociation inhibitor; GDP: guanosine diphosphate; GTP: guanosine triphosphate; GAP: GTPase-activating proteins.

Rho GTPases are regulated by a number of other proteins. They are activated by guanine nucleotide exchange factors, and their activity is accelerated by GTPase-activating proteins (Moon and Zheng, 2003; Rossman et al., 2005). Inactive Rho GTPases are chaperoned by a protein called GDI (guanine nucleotide dissociation inhibitor; Garcia-Mata et al., 2011). However, it is not known whether GDI extracts inactive Rho GTPases from the membrane, or how it coordinates with the other proteins that regulate Rho GTPases. These voids in the field exist partly because currently available probes only track active GTPases. Now, in eLife, Adriana Golding and William Bement (University of Wisconsin-Madison) and Ilaria Visco and Peter Bieling (Max Planck Institute of Molecular Physiology) report how they have adapted a strategy previously used to study Rho GTPases in yeast (Bendezú et al., 2015) and employed it to explore how GDI interacts with Rho GTPases in the frog *Xenopus laevis* and in vitro (Golding et al., 2019).

Golding et al. used two labeling strategies to image two Rho GTPases (Cdc42 and RhoA) in vivo and in vitro. The first strategy consisted of inserting a green fluorescent protein into the middle of the Rho GTPases, so that it would not interfere with the N- or C-termini of the proteins, which are important for activity and localization. In the second approach, they fused a small dye called Cy3 to the proteins. Both of the new tagged versions of Cdc42 and RhoA localized to the correct place and performed their normal roles in the cell.

In wounded *X. laevis* oocytes, tagged Cdc42 (which allows visualization of both the active and the inactive forms of the protein) did not co-localize completely with the active protein. Rather, it extended slightly past the area marked by an activity reporter, suggesting that inactive Cdc42 associates with the plasma membrane. Gradual overexpression of Abr, a protein that inactivates Cdc42 (Vaughan et al., 2011), caused a dose-dependent decrease of active Cdc42 at the wound site, but had a much milder effect on Cdc42 that had been labeled with Cy3. This suggests that both active and inactive Cdc42 are present at the plasma membrane. GDI localization overlapped with the zones of active Cdc42 and RhoA on the membrane, indicating that GDI is involved in localizing active GTPases. Indeed, GDI overexpression suppressed the activity of both proteins, and reduced the total RhoA and Cdc42 at the membrane. Together, these results suggest that GDI helps shape the zones of active and inactive GTPases on the cell membrane.

These results also suggest that GDI regulates active GTPases by extracting them from the membrane. To test this idea, Golding et al. measured GTPase dissociation from in vitro lipid bilayers. GDI was more efficient at extracting the inactive RhoA and Cdc42 from the lipid bilayer, but was also able to extract active forms of the proteins. Golding et al. even identified a GDI mutant that, despite being able to extract active and inactive RhoA from lipid bilayers, could not extract active Cdc42. This GDI mutant could still extract inactive Cdc42 from the membranes, but with slightly lower efficiency than the wildtype inhibitor. Future work will reveal how perturbing GDI extraction of active Cdc42 affects the cytoskeleton of cells. For example, retaining active Cdc42 specifically is likely to cause an imbalance between the assembly of the cytoskeleton and its ability to contract, driven by RhoA, which would slow down wound closure (Benink and Bement, 2005).

The work of Golding et al. prompts a revision of the canonical GTPase cycle, wherein GDI is no longer a passive shuttle for inactive Rho GTPases, but also extracts active GTPases from the membrane (Figure 1; purple arrow). The results also hint at the possibility that GDIs help to ‘prune’ and concentrate the active GTPase zone. Further studies could investigate how GDIs maintain this zone, which must be localized to perform certain tasks, and therefore must depend on mechanisms that control diffusion (Bement et al., 2006).

This work also inspires other questions. First, does the interaction with GDI at the membrane immediately inactivate or extract active Rho GTPases, or can some Rho GTPases escape and activate more effector proteins (Figure 1; green arrow)? Second, given that GDI appears to have higher affinity for GTPases than the guanine nucleotide exchange factor (Li and Zheng, 1997), it remains to be seen how GDI-bound inactive Rho GTPases become activated (Figure 1, dashed arrow). The answers to these questions and more are within our reach, thanks to the work of Golding, Visco, Bieling and Bement.
